# The value of bacterial metagenomic analysis in post-surgical examination of gallstones

**DOI:** 10.1007/s00203-021-02580-4

**Published:** 2021-09-25

**Authors:** T. Ploszaj, M. Brauncajs, M. Traczyk-Borszynska, T. Matyjas, L. Pomorski, T. Wasiak, M. Borowiec

**Affiliations:** 1 Department of Clinical Genetics, Medical University of Lodz, Pomorska 251, 92-213 Lodz, Poland; 2 Department of Microbiology and Medical Laboratory Immunology, Medical University of Lodz, Pomorska 251, 92-231 Lodz, Poland; 3 Department of General and Oncological Surgery, Medical University of Lodz, Pomorska 251, 92-213 Lodz, Poland; 4 Department of Molecular Biology, Medical University of Lodz, Narutowicza 60, 90-136 Łódź, Poland

**Keywords:** Gallstones, Bacteria, Infection, Metagenomic

## Abstract

Gallstone disease is one of the most common causes of hospitalization for gastrointestinal diseases in the world. Recent studies have examined the presence of bacteria in the formation of stones. Our main goal was to determine the overall composition of gallstone microflora. Gallstones were obtained from 24 patients during laparoscopic cholecystectomy from which DNA were extracted. Composition of bacterial flora was evaluated on 16 s rDNA sequencing technique. In the vast majority of samples, bacteria were present, and four groups could be differentiated regarding the flora. Overall composition shows that 87% of the stones were cholesterol/mixed type of gallstone. Additionally, potentially harmful microorganisms (*Streptococcus, Clostridium* and *Kocuria*) that could cause post-surgery complications were identified in several patients. The obtained results indicate that this technique may be useful in analyzing the type of stones and in pinpointing the presence of pathogenic bacteria.

**Supplementary Information:**

The online version contains supplementary material available at 10.1007/s00203-021-02580-4.

## Introduction

One of the most common causes of hospitalization for gastrointestinal diseases is gallstone disease (Russo et al. 2004). Its pathogenesis is a complex process that includes, among others: bilirubin hypersecretion, cholesterol level, diet, bile stasis, metabolic factors and other comorbidities (Lammert et al. 2016). Numerous studies have examined the role of bacteria in the formation of bile. It has been observed that the enzymes produced by some bacteria, such as β-glucuronidase or phospholipases, may favor the formation of bile stones (Nakano et al. 1988, Paumgartner and Sauerbruch 1991). Due to their composition, gallstones are classified into several groups. About 90% of all gallstones in the European population belong to the cholesterol group, where the core of the stones is formed by a combination of cholesterol with calcium salts and glycoproteins (Forrest et al. 2006). The second most common group is the so-called pigmented stones, most often composed of calcium bilirubinate, fatty acids and mucin (Cetta 1991; Vítek and Carey 2003). There are several metagenomic reports indicating that the presence of specific types of bacteria is related to the type of gallstone, i.e. cholesterol or pigmented (Wu et al. 2013; Kose et al. 2018). In this study, we aimed to determine the diversity of the microbiota in random gallstones without specifying their type at the beginning, and to determine the type of particular gallstone based on bacterial composition.

## Materials and methods

### Sample collection

Gallstones were obtained from 24 patients during laparoscopic cholecystectomy and immediately frozen in sterile containers at −20° C. All patients signed informed consent forms for participation in the experiment, which was authorized by the bioethics committee of the Medical University of Łódź, no RNN/339/13/KB.

### DNA isolation

DNA was extracted from gallstones under sterile conditions in a laminar flow hood. Before the powdering process, the stones were washed with sterile 0.9% NaCl. Each stone was then subjected to a grinding process at liquid nitrogen in a SPEX SamplePrep 6770 cryogenic mill. To approximately 100 g of the gallstone powder, 500 µl of 1% SDS was added and incubated on a rotator for 12 h. Lithium chloride was then added to a final concentration of 1.5 M, shaken for another 30 min, and the samples were centrifuged for 5 min. Following this, 10,000 RCF and 400 µl of the supernatant was subjected to isolation using the semi-automatic method in the MagnaPure Compact device (Roche) according to the manufacturer’s instructions.

### Metagenomic library preparation and sequencing

Two techniques of library preparation were used in the study. The first one was based on the Illumina standard protocol which involves the amplification of two variable fragments of the 16 s rRNA gene (V3 and V4) in one long amplicon of approximately 450 bp (Klindworth et al. 2013). The amplification, purification and indexing process were performed according to the manufacturer's standard instructions.

The second method was based on the amplification of three variable fragments of the 16 s rRNA gene (V3, V5 and V6) in separate PCR reactions. Detailed characteristics of the primers are given in Table 1 SI. The amplification reaction was performed using the KAPA HiFi Master Mix polymerase (Kapa Biosystems). After amplification, the amplicons were analyzed in 3% agarose gel electrophoresis. After purification of the amplicons with the NucleoSpin gDNA Clean-up SX kit (Macherey–Nagel), the DNA concentration was determined by fluorimetry using the Qubit 2.0 system (Invitogen). For each sample, three DNA amplicons were pooled at equimolar concentrations. A library was then prepared from the pooled amplicons according to the Meyer & Kircher (Meyer and Kircher 2010) protocol but excluding the enrichment step.

Prepared libraries were sequenced on the Miseq (Illumina) device with a set of reagents for paired reads with 250 cycles.

### Processing of sequence data and bioinformatics

The obtained raw fastq files were processed locally on a Galaxy platform (Afgan et al. 2018). The read quality encoding of fastq files was unified (Blankenberg et al. 2010), and low-quality reads shorter than 200 bp were removed with the Trimmomatic tool (Bolger et al. 2014). Reads were assigned to the OTU using the Kraken 2 algorithm based on a standard reference database based on NCBI taxonomy (Bacteria) (Wood et al. 2019). The results were then visualized and subjected to statistical analysis using the MicrobiomeAnalyst online platform (Chong et al. 2020).

### Confirmation of the presence of Salmonella spp.

To confirm the presence of genetic material of the *Salmonella* genera, we amplified the specific conserved *invA* gene in PCR reaction (Kappa HiFi polymerase) according to the methodology described in the following work (Malmarugan et al. 2011).

## Results

**Fig. 1:**
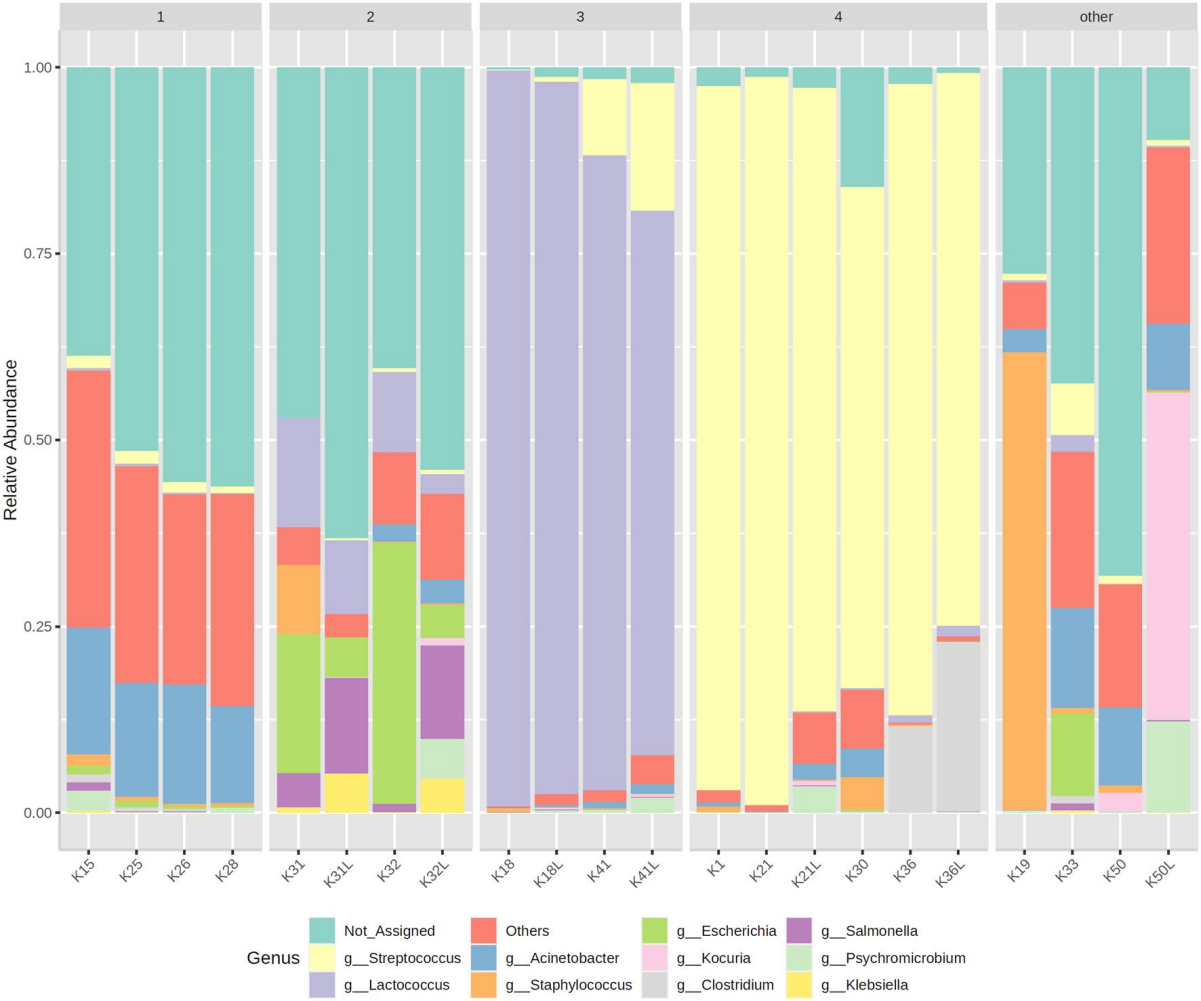
Taxonomic composition of community at *Genus* level: 1—mixed* Acinetobacter, *2*—*mixed* Esherichia, *3* – Lactococcus *dominant,* 4 – Streptococcus *dominant and “other”*.* The results obtained with the long amplicon method (V3–V4) are marked with “L” next to the patient mark

Only 7 results were obtained for the standard method in which 1 long 16 s rRNA gene V3–V4 fragment was amplified, while 15 results were obtained when shorter amplicons were used (62%). The lack of result for some patients corresponds to the lack of PCR product in the agarose gel. After compiling the results for all patients, the gallstone microbiomes were divided into four groups based on their microbiota, and a fifth group consisting of patients with mixed flora. Figure 1 presents a summary of the obtained results according to bacterial genus.

**Fig. 2:**
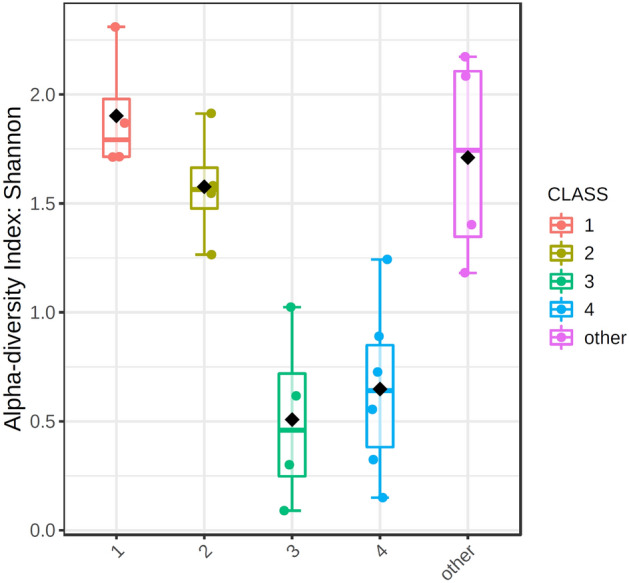
Alpha-diversity measure using Shannon at genus level represented as box-plot. Each box-plot represents the diversity distribution of a one group. 1—mixed* Acinetobacter, *2—mixed* Esherichia, *3 –* Lactococcus *dominant*, *4 –* Streptococcus* dominant and “other”

The alpha-diversity values indicate significant differences between the studied groups (*p* value < 0.001). Figure 2 shows that groups 3 and 4, in which one type of microorganism dominated, have a much lower Shannon diversity index, while the other three groups have a greater diversity maintained at a similar level.

**Fig. 3:**
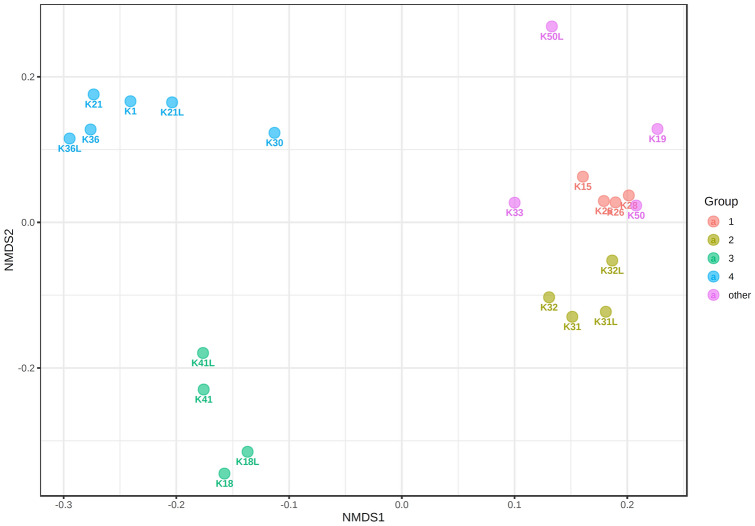
NMDS plot using Jensen Shannon Divergence distance. 1—mixed* Acinetobacter, *2—mixed* Esherichia, *3 –* Lactococcus* dominant*, *4 –* Streptococcus* dominant and “other”

The beta-diversity results also clearly indicate the differences in microbiome composition between the studied groups (PERMANOVA F value 39.005, *p* value  < 0.001). The NMDS (Non-metric MultiDimensional Scaling) chart demonstrates how individuals are focused on into separate groups (Fig. 3).

The clustering results presented on the heatmap (Fig. 1SI) also clearly indicate the differences in the composition of the microbiota in the studied groups due to the frequency of occurrence of particular types of bacteria.

The obtained results indicate the presence of four separate groups of gallstones with similar microbiota compositions at the genera level. The two different methods used to amplify the 16 s rRNA gene mostly showed similar results; however, in a few cases, additional bacteria were identified by the method with one long V3–V4 fragment. Even so, the overall composition of both methods was very similar. A comparison of the results from the two methods is presented in Fig. 2 SI.

## Discussion

### The microbiota of the study groups

Two different methods of library preparation were used. While the first method obtained results for only seven patients, while the second method, with shorter amplicons (~ 150 bp), obtained results for 15 patients. This could be due to the unfavorable conditions in the gallstones caused by the chemical factors present in bile, which degrade DNA molecules.

Although the two methods have very similar results, some individual genera of bacteria such as *Kocuria* or *Klebsiella* were underestimated in the method with shorter amplicons (Fig. 2 SI). These differences could be attributed to the small number of SNPs in the shorter fragment: this would prevent the algorithm from matching the shorter sequence of the 16 s rRNA gene and thus differentiating the samples at the genus level.

The third and fourth groups demonstrated much lower diversity than the other samples (Fig. 2). This is due to the huge prevalence of the *Streptococcaceae* family (above 70%) in these samples. Within this family, there were two types of *Streptococcus* and *Lactococcus* bacteria, which constituted the vast majority of the microbiota in groups three and four. *Streptococci* are facultative anaerobes that generate energy by fermenting carbohydrates to pyruvate with lactic acid as the first metabolite (Lory 2014). This type of bacteria tends to occur in cholesterol-type gallstones (Kose et al. 2018) and it has been indicated that *Lactococcus* occurs more often in people who had gallstones than in healthy people (Wu et al. 2013). Considering the bacteria composition itself, stones from groups 3 and 4 probably belonged to the cholesterol type of gallstones.

Both methods indicated the presence of *Clostridium* in the sample from patient K36. The algorithm assigned the reads to species *Clostridium perfringens*. This bacteria can cause emphysematous cholecystitis and bacteremia, and the beginning of such an infection may have been present in the patient (Gottignies et al. 2010; Atia et al. 2012). However, any inference based only on the analysis of the 16 s rRNA gene fragment to the species level must always be made with some degree of uncertainty.

Two patients from group 2 (K31 and K32) displayed unique and quite diverse microbiota including bacteria from the genus *Escherichia*, *Salmonella* and *Klebsiella*, which were practically absent in other samples. These genera, together with *Enterococcus*, which was absent from our results, are commonly found in pigmented stones (Kose et al. 2018); this suggests that in these two patients, we are dealing with pigmented type of stones. The presence of genetic material of the genus *Salmonella*, which was confirmed by a specific method, suggests that these patients could be asymptomatic carriers of this bacterium. Recent reports indicate that specific properties od *Salmonella spp*. may favor colonization by biofilm formation in gallbladder and on gallstones (Gunn et al. 2014).

A large proportion of the reads in group 2 were unassigned at the genus level; however, this number decreased significantly at the order level. The group is distinguished by a very high percentage of *Enterobacteriales* (Fig. 3 SI) compared to other examined patients. This order includes all the above-mentioned genera (*Escherichia*, *Salmonella*, *Klebsiella,* and *Enterococcus*).

Group 1 is the last to show a consistent and similar composition of the microbiota. At the genus level, most of the reads were assigned to the *Acinetobacter* fraction; this genus may be present, but is not typical for any particular type of gallstones (Wang et al. 2018). Similarly to group 2, although a very large percentage of reads were not assigned at the genus level, many more were at the order level (Fig. 3 SI). These gallstones demonstrated the highest microbiota diversity, and the composition at the order level differed radically from the previously described groups. Interestingly, bacteria of the order *Chitinophagales* were observed, which are not typical of the human digestive tract. This order of bacteria has also been previously identified in the bile of patients (Molinero et al. 2019). It is a relatively poorly studied group of bacteria, the first scientific reports of which date back to 2009 (Lim et al. 2009), and it is difficult to unequivocally define what role they can play in the digestive tract. It is difficult to assign these stones to any particular type based on their high diversity of microbiota; therefore, they can probably best be described as *mixed* gallstones.

The final group of patients had a unique bacterial composition (Fig. 1). Among them, the sample from patient K50 was characterized by a particularly interesting flora, where a very large percentage of the bacteria was *Kocuria*. This type of bacteria has been found to be responsible for acute cholecystitis (Ma et al. 2005). In addition, the sample from patient K19 is unique in that more than 60% of its microbiota is constituted by *Staphylococcus* which has been asociated with acute cholecystitis (Merchant and Falsey 2002). These findings correspond to the clinical picture of the patient, who was urgently admitted to hospital for acute gangrenous cholecystitis with an increased value of lucocytes in the blood. (Table 2 SI).

### Epidemiology

Very few scientific reports have directly compared the composition of the microbiota in randomly selected gallstones; most have focused only on two types of gallstones, i.e. pigmented and cholesterol ones ^9^. The patients marked K31 and K32 form a separate group with gallstones dominated by the bacteria of the *Enterobacteriales*, which was not observed in other patients (Fig. 3 SI). A very similar microbiota composition was identified in the pigment stones in a previous study also based on the NGS technique ^9^.

The remaining patients from groups 3 and 4 had a becteria composition typical of cholesterol stones, or a mixture of different microorganisms (group 1 and other) that made it impossible to assign them to a specific type.

Based on the obtained data, only 2 out of 15 (13%) gallstones can be clearly assigned as pigmented stones; the remaining stones probably belonged to the cholesterol or mixed groups (87%). Such a percentage distribution of the types of stones is consistent with the literature data, which indicates that cholesterol gallstones constitute over 85% of all operated gallstones in developed countries ^20^.

## Conclusion

It is possible to determine the bacterial composition of gallstones, and thus indicate their likely type, using the latest molecular biology methods. Potentially harmful microorganisms (*Streptococcus*, *Clostridium* and *Kocuria*) that can cause postoperative complications were identified in several patients, and their quick and accurate diagnosis can improve treatment. Knowledge on the composition of the gallstone microbiota could help to implement more precise and effective treatment in patients, who reveal the signs of a bacterial infection after surgery.

Our results indicate the presence of *Chitinophagales* bacteria, whose role and influence on the formation of stones is still unknown to this day. In addition, the samples from group 2 were probably mixed stones; these demonstrated considerable microbiota variety and further omics studies are needed to determine the role of bacteria in their formation. We must bear in mind that this work can be only considered as a preliminary report due to the small number of patients tested and the final conclusion can be only made after examining a larger group of subjects.

## Supplementary Information

Below is the link to the electronic supplementary material.

Supplementary file1 (DOCX 1095 kb)

## Data Availability

Not applicable.

## References

[CR1] Afgan E (2018). The Galaxy platform for accessible, reproducible and collaborative biomedical analyses: 2018 update. Nucleic Acids Res.

[CR2] Atia A, Raiyani T, Patel P, Patton R, Young M (2012). Clostridium perfringens bacteremia caused by choledocholithiasis in the absence of gallbladder stones. World J Gastroenterol.

[CR3] Blankenberg D (2010). Manipulation of FASTQ data with Galaxy. Bioinformatics (oxford, England).

[CR4] Bolger AM, Lohse M, Usadel B (2014). Trimmomatic: a flexible trimmer for Illumina sequence data. Bioinformatics.

[CR5] Cetta F (1991). The role of bacteria in pigment gallstone disease. Ann Surg.

[CR6] Chong J, Liu P, Zhou G, Xia J (2020). Using Microbiome analyst for comprehensive statistical, functional, and meta-analysis of microbiome data. Nat Protoc.

[CR7] Forrest K, Welch C, Williams E, Smart H, Lombard M (2006). Investigation of cholesterol gallstone disease. Lancet.

[CR8] Gottignies P, Hossey D, Lasser L, Cherifi S, Devriendt J, De Bels D (2010). Upper gastrointestinal bleeding related to emphysematous cholecystitis due to Clostridium perfringens. Int J Infect Dis.

[CR9] Gunn JS, Marshall JM, Baker S, Dongol S, Charles RC, Ryan ET (2014). Salmonella chronic carriage: epidemiology, diagnosis, and gallbladder persistence. Trends Microbiol.

[CR10] Klindworth A (2013). Evaluation of general 16S ribosomal RNA gene PCR primers for classical and next-generation sequencing-based diversity studies. Nucleic Acids Res.

[CR11] Kose SH, Grice K, Orsi WD, Ballal M, Coolen MJL (2018). Metagenomics of pigmented and cholesterol gallstones: the putative role of bacteria. Sci Rep.

[CR12] Lammert F (2016). Gallstones Nat Rev Dis Primers.

[CR13] Lim JH, Baek SH, Lee ST (2009). Ferruginibacter alkalilentus gen. nov., sp. nov. and Ferruginibacter lapsinanis sp. nov., novel members of the family 'Chitinophagaceae' in the phylum Bacteroidetes, isolated from freshwater sediment. Int J Syst Evol Microbiol.

[CR14] Lory S, Rosenberg E, DeLong EF, Lory S, Stackebrandt E, Thompson F (2014). The Family Streptococcaceae. The Prokaryotes: Firmicutes and Tenericutes.

[CR15] Ma ESK, Wong CLP, Lai KTW, Chan ECH, Yam WC, Chan ACW (2005). Kocuria kristinae infection associated with acute cholecystitis. BMC Infect Dis.

[CR16] Malmarugan S, Velayutham T, Rajeswar J (2011). InvA gene specific PCR for detection of Salmonella from boilers. Veterinary World.

[CR17] Merchant SS, Falsey AR (2002). Staphylococcus aureus cholecystitis: a report of three cases with review of the literature. Yale J Biol Med.

[CR18] Meyer M, Kircher M (2010). Illumina sequencing library preparation for highly multiplexed target capture and sequencing. Cold Spring Harb Protoc.

[CR19] Molinero N (2019). The human gallbladder microbiome is related to the physiological state and the biliary metabolic profile. Microbiome.

[CR20] Nakano T, Yanagisawa J, Nakayama F (1988). Phospholipase-activity in human bile. Hepatology.

[CR21] Paumgartner G, Sauerbruch T (1991). Gallstones—pathogenesis. Lancet.

[CR22] Russo MW (2004). Digestive and liver diseases statistics. Gastroenterology.

[CR23] Vítek L, Carey MC (2003). Enterohepatic cycling of bilirubin as a cause of ‘black’ pigment gallstones in adult life. Eur J Clin Invest.

[CR24] Wang YN, Qi M, Qin C, Hong JB (2018). Role of the biliary microbiome in gallstone disease. Expert Rev Gastroenterol Hepatol.

[CR25] Wood DE, Lu J, Langmead B (2019). Improved metagenomic analysis with Kraken 2. Genome Biol.

[CR26] Wu T (2013). Gut microbiota dysbiosis and bacterial community assembly associated with cholesterol gallstones in large-scale study. BMC Genomics.

