# Supplementary material for: The value of bacterial metagenomic analysis in post-surgical examination of gallstones
Source: Arch Microbiol. 2021 Sep 25;203(10):6323–8. doi: 10.1007/s00203-021-02580-4 (PMC8590668; doi:10.1007/s00203-021-02580-4)
Supplement: Supplementary file 1 — Supplementary file1 (DOCX 1095 kb) [file 203_2021_2580_MOESM1_ESM.docx]

**Table 1**. Primer characteristic 16S rRNA.

| **16S rRNA** | **Primer sequence** | **Length [bp]** | **Annealing temperature** | ***E. coli* position J01695** | **Reference** |
| --- | --- | --- | --- | --- | --- |
| **V3** | F: CCTACGGGRSGCAGCAG  R: ACCGCGGCKGCTGGC | 148-192 | 54°C | 341-357;  517-531 | [^1^](#_ENREF_1) |
| **V5** | F: GGATTAGATACCCBRGTAGTC  R: CCGTCAATTCMTTTRAGTTT | 141-146 | 55°C | 785-805;  907-926 | [^2^](#_ENREF_2) |
| **V6** | F: AAACTYAAAKGAATTGACGGG  R: CGACARCCATGCASCACCT | 152-176 | 50°C | 907-927; 1046-1064 | [^3^](#_ENREF_3) |
| **V3-V4** | F:TCGTCGGCAGCGTCAGATGTGTATAAGAGACAGCCTACGGGNGGCWGCAG  R:GTCTCGTGGGCTCGGAGATGTGTATAAGAGACAGGACTACHVGGGTATCTAATCC | ~450 | 55°C | 341-785 | [^4^](#_ENREF_4) |

1. Hansen MC, Tolker-Nielsen T, Givskov M, Molin S. Biased 16S rDNA PCR amplification caused by interference from DNA flanking the template region. *FEMS Microbiology Ecology.* 1998;26(2):141-149.

2. Lane DJ, Pace B, Olsen GJ, Stahl DA, Sogin ML, Pace NR. Rapid determination of 16S ribosomal RNA sequences for phylogenetic analyses. *Proc Natl Acad Sci U S A.* 1985;82(20):6955-6959.

3. Adler CJ, Dobney K, Weyrich L, et al. Sequencing ancient calcified dental plaque shows changes in oral microbiota with dietary shifts of the Neolithic and Industrial revolutions. *Nature Genetics.* 2013;45:450-455.

4. Klindworth A, Pruesse E, Schweer T, et al. Evaluation of general 16S ribosomal RNA gene PCR primers for classical and next-generation sequencing-based diversity studies. *Nucleic Acids Res.* 2013;41(1):e1-e1.


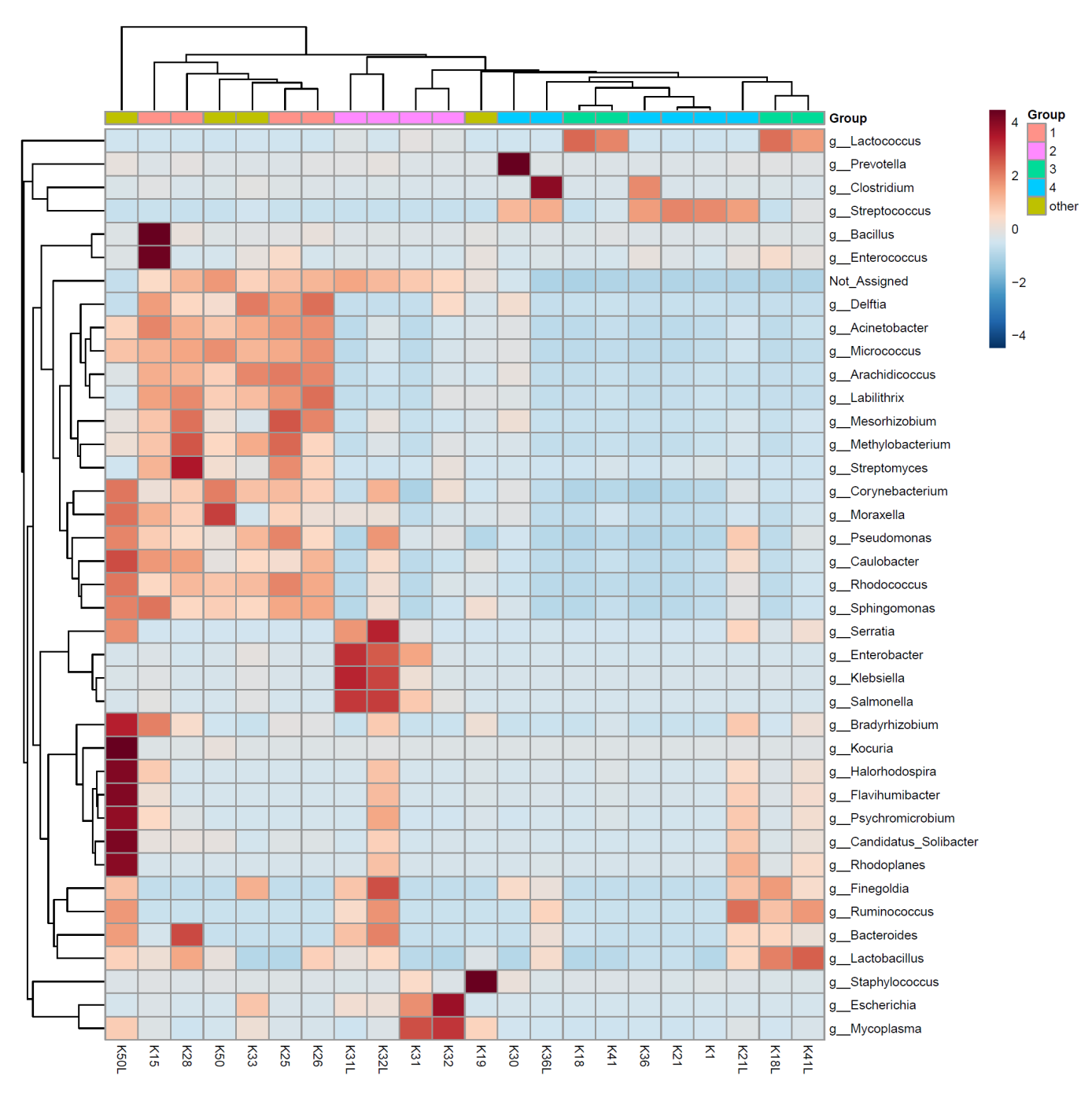


**Figure 1.** Clustering result at genus level shown as heatmap (distance measure: Euclidean; clustering algorithm: average)


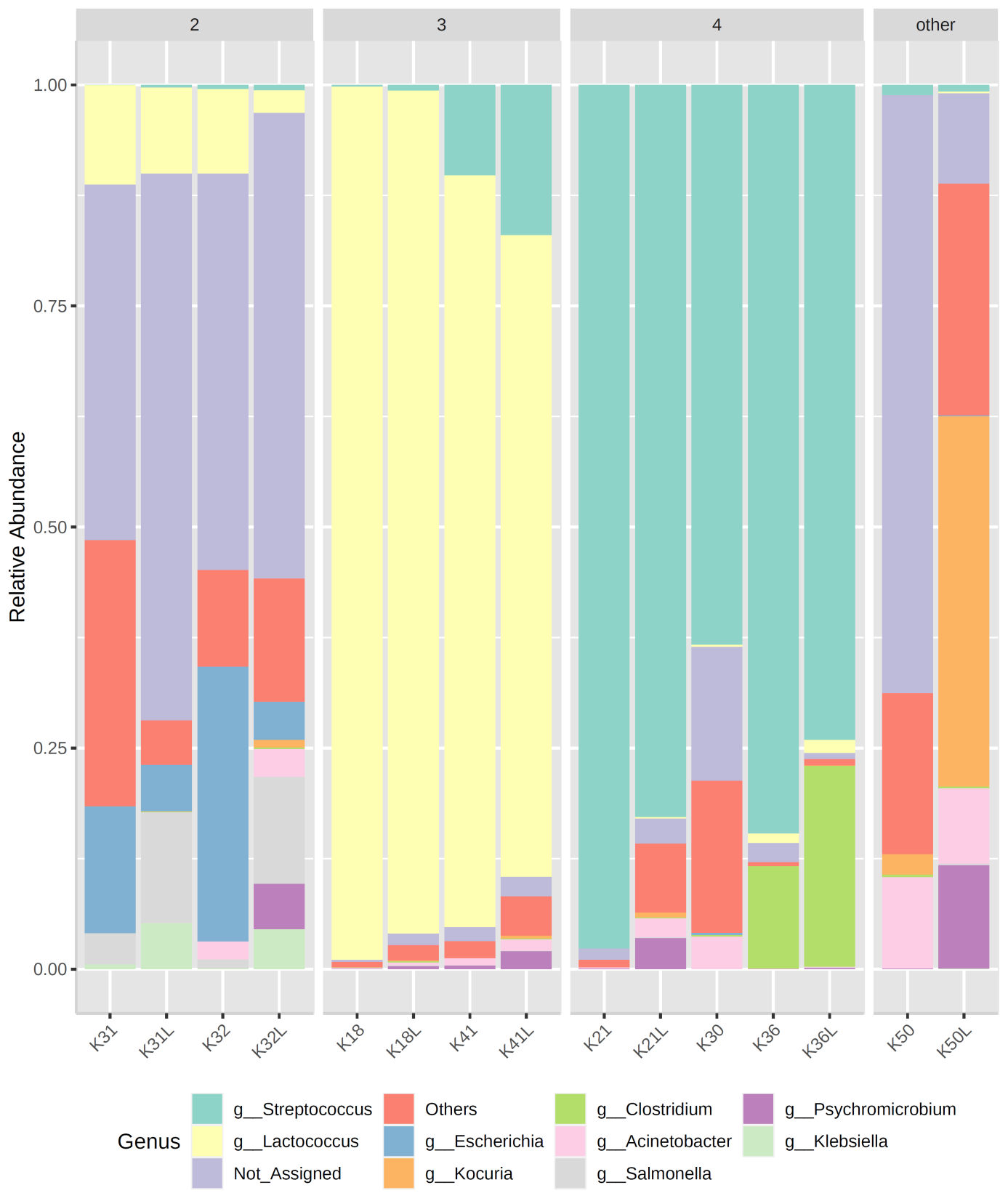


**Figure 2.** Taxonomic composition of community at Genus level for results obtained from different methods used. The results obtained with the long amplicon method (V3-V4) are marked with "L" next to the patient mark.


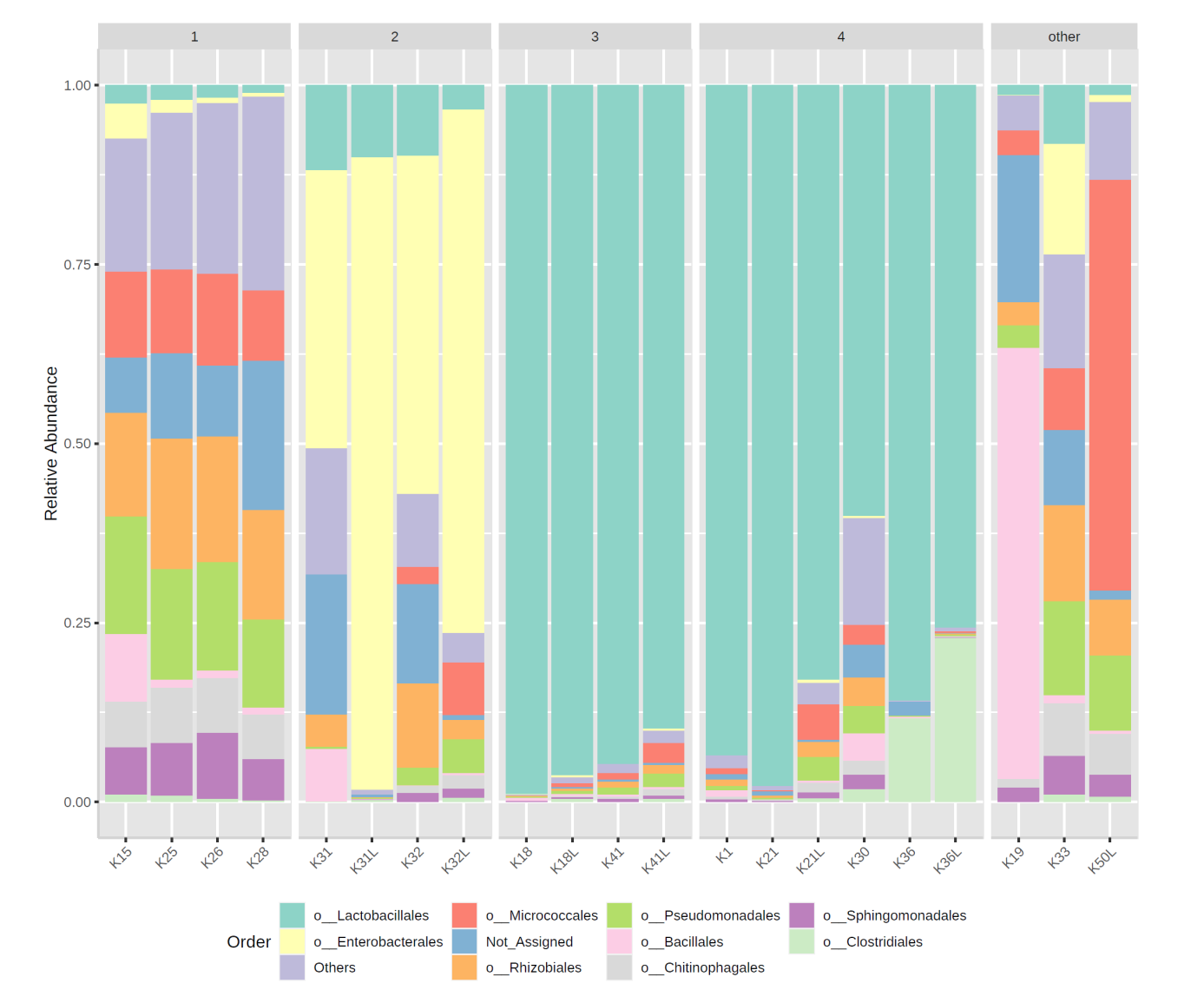


**Figure 3.** Taxonomic composition of community at Order level. The results obtained with the long amplicon method (V3-V4) are marked with "L" next to the patient mark.

**Clinical characteristic of studied population**

The study included 15 patients undergoing scheduled and urgent surgery at the Department of General and Oncological Surgery (Central Clinical Hospital of the Medical University of Łódź) (from September 2014 to March 2017). There were 11 (73.3%) women and 4 (26.7%) men in the study group, aged 29-79, mean 59 years. Among the studied population, 13 (86.7%) patients were admitted electively because of symptomatic cholecystolithiasis, and 2 (13.3%) were admitted urgently with symptoms of acute cholecystitis due to gallstones. Gallstones confirmed radiographically by ultrasound examination of the abdominal cavity. The patients underwent laparoscopic cholecystectomy of the gallbladder.

| **Patient number** | **Age**  **(years)** | **Sex**  **(M - male, F - female)** | **Hospital admission procedure**  **(S-** **scheduled, A - urgent)** | **Leukocytes**  **(thousand/ul)** | **Histopathological diagnosis** |
| --- | --- | --- | --- | --- | --- |
| **K1** | 75 | F | S | 6.9 | Chronic cholelithiasis |
| **K15** | 43 | F | S | 6.4 | Chronic cholelithiasis |
| **K18** | 71 | F | U | 12.6 | Acute pyoderma cholecystitis |
| **K19** | 50 | F | U | 16.8 | Acute gangrenous cholecystitis |
| **K21** | 59 | F | S | 8.7 | Chronic cholelithiasis |
| **K25** | 29 | F | S | 5.9 | Chronic cholelithiasis |
| **K26** | 38 | F | S | 6.1 | Chronic cholelithiasis |
| **K28** | 34 | F | S | 11 | Chronic cholelithiasis |
| **K30** | 63 | F | S | 5.5 | Chronic cholelithiasis |
| **K31** | 79 | M | S | 5.9 | Chronic cholelithiasis |
| **K32** | 68 | M | S | 7.7 | Chronic cholelithiasis |
| **K33** | 72 | M | S | 4.3 | Chronic cholelithiasis |
| **K36** | 65 | F | S | 7.4 | Chronic cholelithiasis |
| **K41** | 67 | M | S | 5.3 | Chronic cholelithiasis |
| **K50** | 70 | F | S | 4.8 | Chronic cholelithiasis |

**Table 2.** Clinical characteristic of patients.
